# Seasonal Dynamics of Leaf Stoichiometry of *Phragmites australis*: A Case Study From Yangguan Wetland, Dunhuang, China

**DOI:** 10.3390/plants9101323

**Published:** 2020-10-06

**Authors:** Dong Liu, Jian Zhang, Asim Biswas, Jianjun Cao, Huanjie Xie, Xuanxuan Qi

**Affiliations:** 1 College of Geography and Environmental Science, Northwest Normal University, Lanzhou 730070, China; 2018222382@nwnu.edu.cn (D.L.); caojj@nwnu.edu.cn (J.C.); 2019222437@nwnu.edu.cn (H.X.); 2019222455@nwnu.edu.cn (X.Q.); 2 School of Environmental Sciences, University of Guelph, 50 Stone Road East, Guelph, ON NIG 2W1, Canada; biswas@uoguelph.ca

**Keywords:** ecological stoichiometry, *Phragmites australis*, season, Yangguan wetland of Dunhuang

## Abstract

Leaf stoichiometry can enhance our understanding of leaf elements’ (C, N and P) concentrations and their corresponding ratios in an ecosystem with seasonal environment changes. This study quantified the seasonal dynamics of leaf stoichiometry of *P. australis* from Yangguan wetland, Dunhuang, China as a case study example. The leaf C concentration (LC) of *P. australis* changed between seasons and was 392.26 (g·kg^−1^), 417.35 (g·kg^−1^) and 392.58 (g·kg^−1^) in spring, summer and autumn, respectively. Leaf N and P concentrations (LN and LP) were 23.49 (g·kg^−1^), and 17.54 (g·kg^−1^) and 5.86 (g·kg^−1^), and 1.00 (g·kg^−1^), 0.75 (g·kg^−1^) and 0.16 (g·kg^−1^), respectively, in the three seasons. The maximum (77.68) and the minimum values (17.00) of LC:LN were observed in the autumn and spring, respectively. Seasonal variations in LC:LP also showed a similar trend, with the greatest value of 3015.91 in autumn and the lowest value of 429.39 in spring. However, the highest (45.67) and the lowest values (24.18) of LN:LP were observed in autumn and summer, respectively, indicating that the growth of *P. australis* was mainly affected by P. Based on these results, it can be concluded that *P. australis* adopted a competition strategy during the early growth stage but took on a defense life strategy at the late growth stage to cope with various environments.

## 1. Introduction

The balance of energy and its interaction with multiple chemical elements in the ecosystem drives ecological stoichiometry [1,2,3]. Carbon (C), nitrogen (N) and phosphorus (P) concentrations are the most critical life elements in cell structure and function [4,5]. The concentration of these elements and their ratios in leaves or leaf stoichiometry reflects the physiological regulation of N and P uptake by the plant, C assimilation [1,6,7], plant growth rate and strategy and other ecological processes [8]. These also reflect the balance of soil nutrient and plant nutrient demand [1,9,10]. The stoichiometric characteristics of leaves are often used as important indicators to evaluate the nutrient utilization strategies of plants and the nutrient limitations of ecosystems [3,11].

As the growth rate of plants change with the seasons to meet variable nutrient levels in different phenological stages, the stoichiometry of C, N and P in leaves also differ [12,13]. For example, C concentration in leaves (LC) of *Tamarix chinensis* increased, while N and P concentrations (LN and LP) increased first and then decreased in the coastal wetland of Laizhou Bay from spring to autumn [1]. Similarly, in coastal wetlands, LN of *Suaeda salsa* decreased significantly, while LC and LP did not change with the season in the Yellow River Delta, China [14]. In Qinling Mountains, LC of *Larix principis-rupprechtii* Mayr showed no significant seasonal change, while LN was highest in the summer and lowest in the autumn while LP decreased significantly from spring to autumn [15]. Seasonal variations in leaf stoichiometry of *Carex brevicuspis* in different altitude gradients of Dongting Lake were also observed. LC decreased significantly at low altitudes, but it decreased at first and then increased at high altitudes from spring to autumn [16]. These clearly showed that the response of plant leaf stoichiometry varies with the season, species and ecosystem, and each ecologically important species must be examined to determine their response [6,13,17,18].

*Phragmites australis* (*P. australis*), a common perennial reed grass, found in temperate and tropical wetlands around the world but more commonly in Europe and Asia, is important for wildlife conservation, shoreline stability and erosion reduction; it is also used for phytoremediation, thatching roofs, as ornamental plants, for food, herbal medicine and making musical instruments. *P. australis* is also very common and monodominant in the arid wetland areas and plays an essential role in maintaining the ecological function of the wetland ecosystem [19,20,21,22,23]. However, with changing climate and pressure from invasive species and other natural factors, *P. australis* has suffered damage and compromised the ecosystem services they provide. Thus, for its ecological importance, it is necessary to characterize and quantify seasonal variations in leaf stoichiometry of *P. australis* for their continued benefits for their ecosystem functions and services. Although leaf stoichiometry of *P. australis* has been studied in many ecosystems [20,24,25], its seasonal response remains unclear. This limit our understanding of the physiological mechanism and nutrient utilization strategy of *P. australis*, especially in arid wetland areas. The overall objective of this study was to examine the seasonal dynamics of *P. australis* on leaf stoichiometry in an arid wetland. Specifically, this study (ⅰ) examined the seasonal variations in LC, LN and LP and their stoichiometric ratios of *P. australis* and (ⅱ) identified the dominant soil factors affecting leaf stoichiometry in an arid wetland from the Yangguan wetland area of Dunhuang, China as a case study. Our hypothesis was that soil water and temperature may be the main factors affecting leaf stoichiometry of *P. australis* since water and energy (heat) drive the primary ecological functions in arid regions [26].

## 2. Results

### 2.1. Variations on Leaf Stoichiometry and Soil Properties with the Seasons

**Figure 1:**
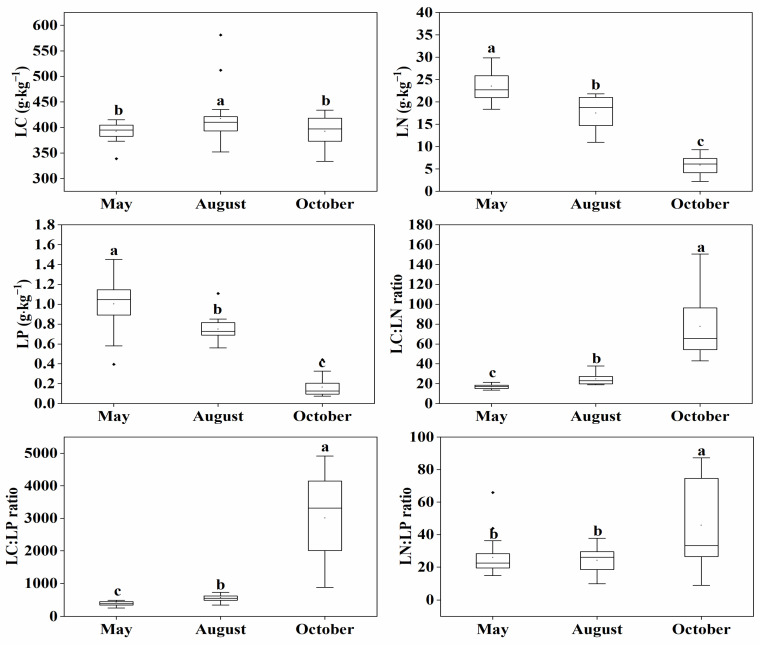
Difference in leaf stoichiometry of *P. australis* among different stages of the growing season. LC, leaf carbon concentration; LN, leaf nitrogen concentration; LP, leaf phosphorus concentration. Different small letters mean a significant difference at the 0.05 level.

The LC, LN and LP and their ratios of *P. australis* (Figure 1) showed significant seasonal variations. LC was the highest (417.35 g·kg^−1^) at the mid-growing season (August), and was significantly higher than that of early (May) and late (October) growing season. Maximum LN and LP (23.49 and 1.0 g·kg^−1^, respectively) were recorded at the beginning of the growing season, and then decreased to the lowest at the late growing season (5.86 and 0.16 g·kg^−1^, respectively), showing a significant downward trend over the growing season. The LC:LN and LC:LP were lowest at the early growing season (17 and 429.39, respectively), and their maximum values were reached at the late growing season (77.68 and 3015.91, respectively). For LN:LP ratio, there was no significant difference between the early and mid-growing season. However, it significantly increased during the late growing season (from 24.18 in Summer to 45.67 in Autumn).

The season had a significant effect on soil organic carbon (SOC), soil nitrate nitrogen (NO_3_^−^-N), soil temperature (ST) and soil nutrient stoichiometric ratios (SOC:STN, SOC:STP, and STN:STP; Table 1) (STN, soil total nitrogen; STP, soil total phosphorus). The SOC was the highest in summer (10.61 g·kg^−1^) and was significantly higher than that in the spring and autumn (5.79 and 6.89 g·kg^−1^, respectively). The NO_3_^−^-N showed an increasing trend with the growing season, with a significantly higher amount in autumn (11.29 mg·kg^−1^) than that of the spring (6.34 mg·kg^−1^). The ST varied significantly with the seasons, with the highest value in summer (22.67 ℃) and the lowest value in winter (12.5 ℃).

### 2.2. Relationship Between Leaf Stoichiometry and Soil Properties

**Figure 2:**
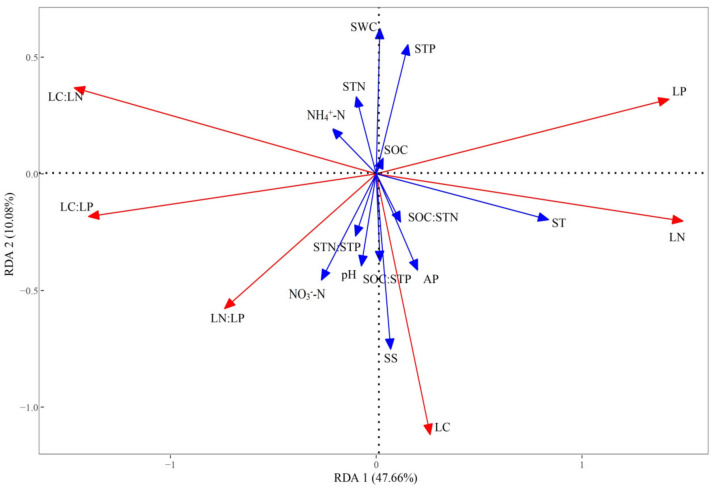
Redundancy analysis (RDA) ordination for leaf stoichiometry of *P. australis* and soil environmental factors. SOC, soil organic carbon; STN, soil total nitrogen; STP, soil total phosphorus; AP, soil available phosphorus; NH_4_^+^-N, soil ammonium nitrogen; NO_3_^−^-N, soil nitrate nitrogen; SWC, soil water content; ST, soil temperature; SS, soil salt; LC, leaf carbon concentration; LN, leaf nitrogen concentration; LP, leaf phosphorus concentration.

The Redundancy analysis (RDA) showed that soil properties (SOC, STN, STP, SOC:STN, SOC:STP, STN:STP, AP, NO_3_^−^-N, NH_4_^+^-N, SWC, SS, ST and pH) (AP, soil available phosphorus; SS, soil salt; NH_4_^+^-N, soil ammonium nitrogen; SWC, soil water content) explained 62.88% of the total variation in the data, with axes 1 and 2 explaining 47.66% and 10.88%, respectively (Figure 2). The species-environment relationship of soil variables in axis 1 and axis 2 accounted for 93.10% of the total variance. This means that ST, SWC, SOC, AP, NO_3_^−^-N and SS were the main factors influencing leaf stoichiometry of *P. australis* (Table 2).

## 3. Discussion

### 3.1. Seasonal Fluctuations of Leaf Stoichiometry

A previous study by Liu et al. [27] indicated that the random effect of sample plots could interfere with the independence of samples. Based on the linear mixed model, the random effect of plots was not significant but fixed effect of season was significant (Table 3). Phenology is an essential characteristic of plant adaptation and evolution in the environment, and it involves an unbalanced distribution of nutrients in functional metabolism [28]. During the early growth stage, the cell division rate gradually increases requiring selective absorptions of N and P, and a portion of the stored nutrients in the roots were transported to the leaves of *P. australis*, resulting in higher LN and LP [15,29,30]. *P. australis* was in rapid growth period in August, leaf photosynthesis was enhanced and LC was accumulated rapidly, but LN and LP diluted with the rapid increase of biomass, leading to a significant decrease in LN and LP [14,31]. During the late growing stage, with leaves senescence and nutrients return to roots, LN and LP decreased again [32,33]. Also, LC was reduced significantly as more carbohydrates were distributed to the roots [15].

The C:N:P ratio reflects a balance between competition and defensive life strategies [1]. In the present study, LN and LP decreased significantly from early to the mid-growth stage, LC increased significantly, and the leaf had a high photosynthetic rate. These indicated that *P. australis* undertook a competitive strategy [34,35]. The N and P are essential nutrients for plant photosynthesis such as protein synthesis, enzyme transport and C assimilation, and can directly participate in a series of metabolic processes [7]. Plants in the study area suffered from long-term salt stress, and additional photosynthetic storage products could have been rapidly synthesized by increasing leaf nutrient content. This would have played a crucial role in osmotic protection and regulation [26]. At the late growing stage, allocating more carbohydrates to roots resulted in conserving resource and energy, and thus, the plant adopted a defensive life strategy [31].

### 3.2. The Dominant Environmental Factors Influencing Leaf Stoichiometry

RDA analysis showed that main soil factors affecting the stoichiometry of *P. australis* leaves were SS, ST, SWC, SOC, NO_3_^−^-N and AP, partly supporting our hypothesis. In an arid wetland ecosystem, others [7,36] also found that salt (water) is one of the most important ecological factors that restrict plant growth and has considerable significance for the life history strategy of desert plants to strengthen the effective utilization of resources under environmental stress.

Among these dominate factors, SS was positively correlated to LC and LN, which was in line with other studies [37,38]. Sun et al. [37] found that optimum SS can enhance carbon fixation of halophytes. This may be attributable to a significant increase in photosynthesis, or an increase in solute uptake to induce cell expansion to maintain the pressure potential in plant tissues. Furthermore, the concentrations of amino acids and N in plants with the increase of SS stress from the high concentration of NaCl can reduce the synthesis rate of proteins in plants, accelerating the hydrolysis of stored protein and causing the accumulation of ammonia in the body [1,39]. In the present study, SWC was negatively related to LC, as found by Lin et al. [40] because more SWC was used by leaf transpiration and thus, caused soil desiccation [41]. However, at a global scale, Ordoñez et al. [42] reported that SWC had no effect or negative effect on leaf nutrient concentration. This suggested that the effects of SWC on leaf stoichiometry may be ecosystem type and plant species dependent. The SWC was significantly negatively correlated with LN:LP, but it was not significantly related to LN or LP (Table 4), indicating that SWC had a different effect on the leaf element’s concentration and their stoichiometric ratios. More exploration is needed to understand the reasons for these effects.

Soil NO_3_^−^-N was negatively related to LN and LP (Table 4), and the reason may be attributed to the conversion of the NO_3_^−^-N into organic nitrogen in leaves; besides, the leaves need to spend more ATP in assimilating NO_3_^−^-N [43]. The AP was positively correlated with LP because most plants obtain P through their roots and thus, AP appears to have been a controlling factor for LP in our study [25,44,45]. However, the reason for the positive correlation with LC may be that P is necessary for photosynthesis and can be directly involved in photophosphorylation and C assimilation, indicating that soil AP plays an important role in leaf photosynthesis [7].

The ST can both affect the plant physiological processes and stimulate soil enzyme activity as well as the production and degradation of biomass [36,46,47]. Lu et al. [48] found that the alternate freezing and thawing accelerated decomposition of plant debris and their return to surface soils. The ST was the highest in the summer and stimulated the activities of decomposers [49], leading to an increase of SOC and STN (Table 5), which in turn played a positive role in the rapid growth stage of *P. australis*. In the present study, ST was positively correlated with LC, LN and LP (Table 4), as was also reported by others [4,24,50,51]. The reason for this may be that as plants within the arid wetland areas have a short growing season, the fact that leaf nutrients increase with the increase in ST could balance intracellular osmotic pressure and strengthen the protection of leaf internal moisture [36,44,52].

### 3.3. Stoichiometric Limiting Elements in Leaves

LN:LP ratio was considered as an index to judge the nutrient supply status of the environment to plant growth [1,10]. For example, Koerselman and Meuleman [9] suggested that, when LN:LP > 16, plants showed P-limited. A value 14 < LN:LP < 16 meant an equal limitation of N and P on plant growth, when LN:LP < 14, plants were restricted by N in wetland ecosystems. Our findings showed that average LN:LP >16 in *P. australis* and across the growing seasons, indicating that the growth of *P. australis* was limited by P. This was consistent with the results of tropical and subtropical freshwater wetlands [53,54]. Growth of *P. australis* limited by P can also confirm the fact that, in the study area STP was only 0.4 g·kg^−1^, lower than that of the average value of the Chinese mainland (0.65 g·kg^−1^) [55].

## 4. Materials and Methods

### 4.1. The Study Area

**Figure 3:**
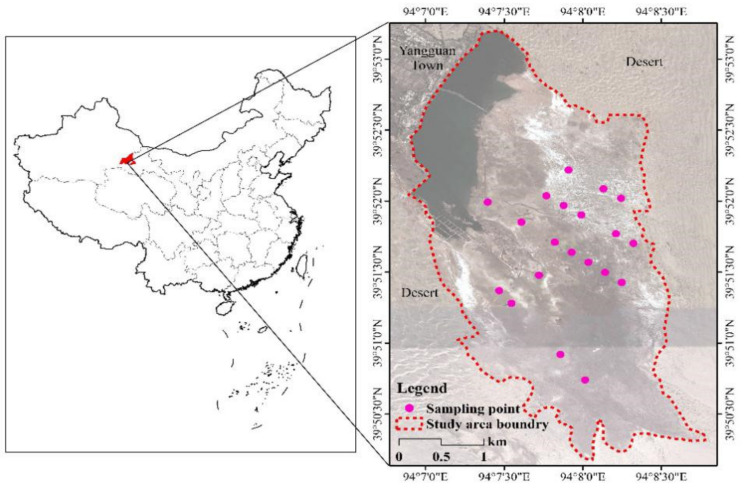
Location of sampling points in Yangguan wetland.

The Yangguan wetland (93.88–94.28° E, 39.65–40.08° N), belongs to Dunhuang Yangguan National Nature Reserve (DYNNR), and it is located in Yangguan Town, Dunhuang city, the westernmost point of the Gansu Province, China (Figure 3). The study area is an oasis extending westward in the Qilian Mountains but is mainly surrounded by desert, covering an area of 882 km^2^ and altitude ranging from 1150 to 1500 m. Its water source is from ice and snow melting water and precipitation in the mountainous area, and it is relatively stable [56]. The annual average temperature is 9.3 ℃, and the annual average precipitation is 36.9 mm, with means annual evaporation of 2465 mm. The main vegetation type in the area is temperate desert vegetation, with *P. australis* being the dominant species along with *Leymus chinensis, Salicornia salsa, Lycium ruthenicum, Scorzonera austriaca* and *Glaux maritima* and some associated species. The basic soil properties from May to October are presented in Table 5 (more details in Section 2).

### 4.2. Sampling and Measurement

According to the topographic features of the wetland, 20 plots (30 × 30 m) were identified along the vertical direction of variations (Figure 3). In the study area, *P. australis* usually bud in April, grow rapidly during July to early September, then the growth slows down in late September to early October, and leaves senesce and fall off by the middle of October. In 2018, during mid-May, mid-August, and mid-October, healthy leaves from these plots were collected. After each leaf sampling, soil samples at depths of 0–20, 20–40 and 40–60 cm were collected using standardized collection schemes [1]. All collected leaf samples were dried at 65 ℃ for 48 hours to constant weight using an oven in the laboratory. LC and soil organic carbon (SOC) were measured using the wet oxidation with the K_2_Cr_2_O_7_-H_2_SO_4_ Walkley-Black oxidation method [57]. LN and soil total nitrogen (STN) were determined using the Kjeldahl method. Soil total phosphorus (STP), soil available P (AP) and LP were measured using Molybdenum-Antimony colorimetry [58]. Soil ammonium nitrogen (NH_4_^+^-N) and nitrate nitrogen (NO_3_^−^-N) were measured using a SmartChen 200 element analyzer (AMS Rome., ITA). Soil water content (SWC) was determined by drying soil samples in an oven at 105 ℃ to a constant mass. Soil pH was measured by a Sartorius PB-10 pH meter (Sartorius., Beijing, China). Soil salt (SS) was measured in solution of soil and distilled water (1:5, w/v) using an FE38 conductivity meter (Mettler Toledo Co., Ltd., Shanghai, China). The soil temperature (ST) was measured using a TH6 humidity and temperature data logger (Dickson., Shenzhen, China) for a week in each growth stage for each soil layer (i.e., 0–20, 20–40 and 40–60 cm).

### 4.3. Data Analysis

The relationship between leaf stoichiometry of *P. australis* and soil properties was analyzed with a Spearman rank correlation. As our experimental set-ups were dealing with non-independent data or pseudoreplicates (multiple plots being the random factor, and different seasons being the fixed factor), and random effects of sample plots were tested using a linear mixed-effect model of statistical program R to determine sample independence [27]. One-way analysis of variance (ANOVA) was used to determine seasonal variations in soil properties. Significance analysis was performed using the Tukey post-hoc test. Redundancy analysis (RDA) was performed with a subset of environmental variables to assess the relative impact of abiotic factors on plant leaf stoichiometry, using explanatory environmental variables (SOC, STN, STP and their corresponding ratio, pH, SS, SWC, ST, AP, NO_3_^-^-N and NH_4_^+^-N) as regression covariates [2]. Detrended correspondence analyses of the datasets were undertaken before performing RDA to ensure that gradient lengths fit a linear model [3]. However, it should be noted that when there is no redundancy between the change of response matrix and the change of interpretation matrix, caution should be exercised. All data were logarithmically transformed before parameter testing and passed the homogeneity of variance test.

## 5. Conclusions

Leaf nutrient concentrations and their ratios of *P. australis* exhibited significant seasonal variations in Yangguan wetland area, Dunhuang, China. At the beginning of the growing season, LN and LP concentrations of *P. australis* were high, while the accumulation rate of LC is relatively higher than that of LN and LP, and LN and LP decreased significantly at later stages. In order to strengthen the effective utilization of resources and resist salinity stress, two different strategies, including a competition strategy and a defense strategy were adopted in the growing seasons of *P. australis*, with the former being adopted at the beginning of the growing season and the latter being adopted later in the growing season.

Soil properties contributed significantly to leaf stoichiometry of *P. australis*, and ST, SWC, SOC, AP, NO_3_^−^-N and SS were the key factors affecting leaf stoichiometry during the whole growing season. *P. australis* was limited by P in the study area. This study was carried out on a relatively small scale, and, thus, it is not clear if the conclusion applies to the entire arid region. This needs to be further investigated on a larger scale.

## Figures and Tables

**Table 1:** Effects of season on soil properties.

	Factor	Season		
Properties		*F*	*P*	*df*
SOC (g·kg^−1^)		19.92	**0.000**	2
STN (g·kg^−1^)		2.27	0.133	2
STP (g·kg^−1^)		1.58	0.214	2
SOC:STN		20.05	**0.000**	2
SOC:STP		21.65	**0.000**	2
STN:STP		3.68	**0.031**	2
AP (mg·kg^−1^)		0.48	0.622	2
NH_4_^+^-N (mg·kg^−1^)		2.46	0.094	2
NO_3_^−^-N (mg·kg^−1^)		7.47	**0.001**	2
SWC (%)		0.07	0.932	2
ST (°C)		329.51	**0.000**	2
pH		0.76	0.472	2
SS (g·kg^−1^)		10.76	0.474	2

SOC, soil organic carbon; STN, soil total nitrogen; STP, soil total phosphorus; AP, soil available phosphorus; NH_4_^+^-N, soil ammonium nitrogen; NO_3_^−^-N, soil nitrate nitrogen; SWC, soil water content; ST, soil temperature; SS, soil salt. F, Fisher test (joint hypotheses test); P, probability value; df, degree of freedom. Significant values are in bold.

**Table 2:** The explained variance of soil properties variable and their significant analysis in the first two axes in redundancy analysis (RDA) ordination.

Soil Properties Variable	RDA 1	RDA 2	R^2^	*P* *	Explains (%)	*F*	*P* **
SOC (g·kg^−1^)	0.162	−0.987	0.12	0.03	10	12.4	**0.002**
STN (g·kg^−1^)	0.355	−0.935	0.09	0.088	0.5	0.7	0.56
STP (g·kg^−1^)	−0.896	−0.444	0.04	0.332	0.5	0.7	0.568
SOC:STN	−0.258	−0.966	0.03	0.378	0.5	0.7	0.546
SOC:STP	0.515	−0.857	0.07	0.154	3.3	4.6	**0.01**
STN:STP	0.763	−0.647	0.05	0.279	0.4	0.6	0.664
AP (mg·kg^−1^)	−0.539	0.842	0.21	**0.002**	3.6	4.8	**0.012**
NH_4_^+^-N (mg·kg^−1^)	0.993	−0.115	0.04	0.273	0.4	0.6	0.618
NO_3_^−^-N (mg·kg^−1^)	0.993	0.115	0.14	**0.017**	0.5	0.8	0.53
SS (g·kg^−1^)	−0.307	0.952	0.24	**0.001**	0.9	1.3	0.244
pH	0.717	0.697	0.03	0.376	1.8	2.6	0.052
SWC (%)	0.116	−0.993	0.23	**0.001**	8	8.3	**0.002**
ST (°C)	−0.983	0.181	0.64	**0.001**	36.6	33.4	**0.002**

SOC, soil organic carbon; STN, soil total nitrogen; STP, soil total phosphorus; AP, soil available phosphorus; NH_4_^+^-N, soil ammonium nitrogen; NO_3_^−^-N, soil nitrate nitrogen; SWC, soil water content; ST, soil temperature; SS, soil salt. * indicates the environmental factor is significantly related to the two axes. ** indicates the variance of each environmental factor significantly contributed to the total variance. Significant values are in bold.

**Table 3:** Linear mixed effect model results of the effects of season and plot on leaf stoichiometry of *P. australis*.

Variable	Season (Fixed Effects)				Plot (Random Effects)		
	Standard Error	df	*t*-Value	*p*-Value	df	L-Ratio	*p*-Value
LC	7.9	38	49.65	**0.000**	3	3.43	0.0641
LN	0.71	38	32.9	**0.000**	3	5.27	0.9998
LP	0.04	38	25.8	**0.000**	3	1.46	0.9999
LC:LN	4.25	38	4.0	**0.0003**	3	5.28	0.9998
LC:LP	174.58	38	2.46	**0.018**	3	1.02	0.9997
LN:LP	4.04	38	6.44	**0.000**	3	0.66	0.4174

LC, leaf carbon concentration; LN, leaf nitrogen concentration; LP, leaf phosphorus concentration. df, degree of freedom; L-Ratio, likelihood ratio. Significant values are in bold.

**Table 4:** The relationship between the dominant factors from the RDA and leaf stoichiometry of *P. australis*.

Leaf Stoichiometry	LC (g·kg^−1^)	LN (g·kg^−1^)	LP (g·kg^−1^)	LC:LN	LC:LP	LN:LP
SS (g·kg^−1^)	0.501 **	0.311 **	−0.001	−0.258*	0.048	0.412 **
SWC (%)	−0.234 *	−0.214	0.076	0.191	−0.098	−0.411 **
ST (°C)	0.310 **	0.795 **	0.764 **	−0.781 **	−0.737 **	−0.273 *
SOC (g·kg^−1^)	0.111	−0.119	0.057	0.137	−0.047	−0.255 *
NO_3_^−^-N (mg·kg^−1^)	0.248 *	−0.242 *	−0.349 **	0.281 *	0.374 **	0.285 *
AP (mg·kg^−1^)	0.225 *	0.092	0.329 **	−0.311 **	−0.071	0.275 *

SOC, soil organic carbon; AP, soil available phosphorus; NO_3_^−^-N, soil nitrate nitrogen; SWC, soil water content; ST, soil temperature; SS, soil salt; LC, leaf carbon concentration; LN, leaf nitrogen concentration; LP, leaf phosphorus concentration. * *p* < 0.05, and ** *p* < 0.01.

**Table 5:** Soil nutrient contents, temperature, moisture, salinity and pH across the season within the study area.

Parameter	May	August	October
SOC (g·kg^−1^)	5.79 ± 0.4b	10.61 ± 0.57a	6.89 ± 0.5b
STN (g·kg^−1^)	0.46 ± 0.03a	0.55 ± 0.03a	0.49 ± 0.04a
STP (g·kg^−1^)	0.43 ± 0.01a	0.39 ± 0.02a	0.38 ± 0.02a
SOC:STN	12.96 ± 0.71b	19.71 ± 0.99a	14.25 ± 0.55b
SOC:STP	13.71 ± 0.88c	29.93 ± 3.21a	18.07 ± 1.18b
STN:STP	1.09 ± 0.07b	1.55 ± 1.66a	1.31 ± 0.1ab
AP (mg·kg^−1^)	4.67 ± 0.57a	4.95 ± 0.73a	4.02 ± 0.59a
NH_4_^+^-N (mg·kg^−1^)	9.76 ± 0.95a	11.24 ± 0.63a	11.63 ± 0.77a
NO_3_^−^-N (mg·kg^−1^)	6.34 ± 0.94b	9.53 ± 1.37ab	11.29 ± 1.17a
SWC (%)	25.31 ± 0.03a	22.18 ± 0.02a	22.32 ± 0.02a
ST (°C)	18.09 ± 0.63b	22.67 ± 0.52a	12.5 ± 0.4c
SS (g·kg^−1^)	4.6 ± 0.57a	6.58 ± 0.95a	5.5 ± 0.93a
pH	8.38 ± 0.04a	8.42 ± 0.06a	8.46 ± 0.04a

SOC, soil organic carbon; STN, soil total nitrogen; STP, soil total phosphorus; AP, soil available phosphorus; NH_4_^+^-N, soil ammonium nitrogen; NO_3_^−^-N, soil nitrate nitrogen; SWC, soil water content; ST, soil temperature; SS, soil salt. Row-wise non-matching letters indicate a significant difference among seasons (*p* < 0.05).
